# CMV quantitative PCR in the diagnosis of CMV disease in patients with HIV-infection – a retrospective autopsy based study

**DOI:** 10.1186/1471-2334-7-127

**Published:** 2007-11-06

**Authors:** Arne B Brantsaeter, Mona Holberg-Petersen, Stig Jeansson, Anne K Goplen, Johan N Bruun

**Affiliations:** 1 Department of Infectious Diseases, Ullevaal University Hospital and Faculty of Medicine, University of Oslo, Oslo, Norway; 2 Department of Internal Medicine, Asker and Baerum Hospital, Rud, Norway; 3 Department of Microbiology, Ullevaal University Hospital and Faculty of Medicine, University of Oslo, Oslo, Norway; 4 Department of Pathology, Ullevaal University Hospital and Faculty of Medicine, University of Oslo, Oslo, Norway

## Abstract

**Background:**

Patients with advanced HIV infection at the time of diagnosis and patients not responding to antiretroviral therapy are at risk of cytomegalovirus (CMV) disease. Earlier studies of patients with HIV infection have demonstrated that the diagnosis is often first made post-mortem. In recent years new molecular biological tests have become available for diagnosis of CMV disease. Although clinical evaluation of tests for diagnosis of CMV disease in HIV-infected individuals is suboptimal without autopsy, no results from such studies have been published. The aim of this study was to explore the diagnostic utility of CMV quantitative polymerase chain reaction (PCR) in plasma from HIV and CMV seropositive patients who died during the period 1991–2002 and in whom autopsy was performed.

**Methods:**

Autopsy was performed in all cases, as part of routine evaluation of HIV-infected cases followed at Ullevaal University Hospital. Of 125 patients included, 53 had CMV disease, 37 of whom were first diagnosed at autopsy. CMV disease was diagnosed either by ophthalmoscopic findings typical of CMV retinitis, biopsy or autopsy. One or two plasma samples taken prior to the first diagnosis of CMV disease (alive or at autopsy) or death without CMV disease were analysed by CMV quantitative PCR. Sensitivity, specificity, positive and negative predictive values were calculated for different CMV viral load cut-offs and according to detection of viraemia in one versus two samples.

**Results:**

Twenty-seven of 53 patients with CMV disease (51%) and 10 of 72 patients without CMV disease (14%) had detectable viraemia in at least one sample. Sensitivity and negative predictive value (NPV) of the test, maximised with a cut-off at the test's limit of detection of CMV viraemia (400 copies/mL), were 47% and 70%, respectively. With cut-off at 10 000 copies/mL, specificity and positive predictive value (PPV) were 100%. With a requirement for CMV viraemia in two samples, specificity and PPV were 100% in patients with CMV viraemia above the limit of detection.

**Conclusion:**

Our results indicate that quantitative CMV PCR is best used to rule in, rather than to rule out CMV disease in HIV-infected individuals at high risk.

## Background

Although the incidence of cytomegalovirus (CMV) disease in HIV-infected patients has declined sharply after the introduction of highly active antiretroviral therapy (HAART) [1], many patients are still at risk of CMV disease [2,3]. Clinical features associated with CMV disease are, with the exception of CMV retinitis, unspecific. In cases with extra-ocular disease, histopathological findings typical of CMV are required for verification of the diagnosis.

Earlier studies, not using modern molecular biological methods for CMV disease, demonstrated that CMV disease was under-diagnosed in HIV-infected patients, and that the diagnosis was often first made at autopsy [4-10]. Newer diagnostic tests may improve the detection rate of CMV disease in this patient population. French guidelines recommend both ophthalmocsopy and testing for CMV viraemia by PCR every three months in patients with CD 4 cell counts below 50–100/mm^3^, whereas U.S. guidelines focus on the early clinical recognition of CMV disease [11,12].

A variety of microbiological tests for detection of CMV DNA in blood [3,13-27] and cerebrospinal fluid [28] have been used in the diagnosis of CMV disease. Previous studies have demonstrated that detection of CMV DNA in blood by PCR is a risk factor for subsequent development of CMV disease. Sensitivity, specificity, positive and negative predictive value of these tests have shown great variation [13,18-20,23,26,27]. In one recent study, plasma CMV viraemia was not found to be associated with later development of CMV disease, but was predictive of death [29].

Because diagnosis of CMV disease is often first made at autopsy, sensitivity and negative predictive value of diagnostic tests may be overestimated, and specificity and positive predictive value may be underestimated if autopsy results are not available. Furthermore, by using a quantitative method it may be possible to improve specificity and positive predictive value of CMV detection. However, to our knowledge, no previous studies have related the results of CMV quantitative PCR to autopsy findings. The objective of this study was to explore the diagnostic utility of CMV quantitative PCR in plasma samples from a group of HIV infected patients who died during the study period and in whom autopsy was performed.

## Methods

Patients included in this retrospective study were HIV and CMV seropositive adolescents and adults who died during the period 1991–2002 while they were followed up at Ullevaal University Hospital. A total of 125 patients were included. Patients were generally not screened for CMV disease, even with low CD 4 cell counts, but fundoscopy or biopsy of relevant organs was performed on the basis of suggestive clinical signs and symptoms. Autopsy was performed in all cases as part of routine practice in the evaluation of HIV-infected cases who died at Ullevaal University Hospital, unless this was objected to by relatives. Patient characteristics are shown in Table 1.

**Table 1:** Characteristics of 125 patients included in the study

Characteristic	Value	
	CMV disease	Without CMV disease
	
	N = 53 (%)	N = 72 (%)
Risk category		
Men who have sex with men	32/53 (60)	30/72 (42)
Intravenous drug user	9/53 (17)	30/72 (42)
Other	10/53 (19)	11/72 (15)
Unknown	2/53 (4)	1/72 (1)
		
Age (median) at time of death, years	39	41
		
Sex		
Male	46/53 (87)	58/72 (81)
Female	7/53 (13)	14/72 (19)
		
CD4 cell count*		
Median count, cells/mm^3^	17	69
<100 cells/mm^3^	47/53 (89)	41/72 (57)
<50 cells/mm^3^	43/53 (81)	32/72 (44)
		
Highly active antiretroviral therapy*	7/53 (13)	21/72 (29)
		
Plasma HIV RNA level*		
Tested	10/53 (19)	35/72 (49)
Median among tested, copies/mL blood	305 000	35 000
HIV RNA < 400 copies/mL blood	0/10 (0)	10/35 (29)
HIV RNA < 50 copies/mL blood	0/10 (0)	5/35 (14)

Data shown are number of subjects per no. of subjects with available data

* at time of last sample prior to diagnosis of CMV disease or death without CMV disease

A full autopsy including neuropathological examination was performed in every case. Paraffin-embedded sections were routinely stained with haematoxylin-eosin (HE), and confirmatory immunohistochemistry for CMV was performed in cases where microscopy of HE stained biopsies were inconclusive. CMV disease was verified by demonstration of characteristic cytomegalocytes with inclusions in histopathological samples by light microscopy. However, ante-mortem diagnosis of CMV retinitis was defined as typical ophthalmoscopic findings in patients examined by an experienced ophthalmologist.

Among 53 patients diagnosed with CMV disease, sixteen patients were first diagnosed before death. Of patients diagnosed alive, twelve had retinitis and four had biopsy verified gastrointestinal infection (colitis 2, oesophagitis 1, stomatitis 1) as their first end-organ manifestation of CMV disease. Except for two patients diagnosed with retinitis alive, all had CMV disease confirmed in one or several organs at autopsy. Thirty-seven cases were first diagnosed at autopsy. In these patients the number of CMV end-organ manifestations at autopsy were: adrenalitis 26, pneumonitis 22, encephalitis 16, retinitis 14, gastrointestinal infection 14 (oesphagitis 11, gastritis 1, small intestine infection 1, colitis 1), and other end-organ disease 11 (prostate gland 5, ovaries 2, uterus 1, epididymis 1, lymph node 2, pancreas 5, spleen 3, liver 3, kidney 4, myocardium 1, thyroid gland 4, vascular endothelium 1). Twenty-patients first diagnosed at autopsy had more than one organ manifestations of CMV disease.

Only twenty-eight patients received HAART at the time of the last plasma sample tested for CMV DNA, either before the first diagnosis of CMV disease (alive or at autopsy), or before death in patients never developing CMV disease. This is because the majority of plasma samples were taken prior to the introduction of HAART in 1996. Seven cases with CMV disease (diagnosed 1997–2002) were on HAART when the last plasma sample was taken and later analysed with CMV PCR. However, HIV viral load was high in all, ranging from 17000 – >750 000 copies/mL. Two of these seven cases had CMV retinitis diagnosed alive, whereas the remaining five were first diagnosed with CMV at autopsy.

Plasma aliquots were routinely collected and stored at -20°C or colder during the study period, as part of clinical follow-up of patients. From cases with CMV disease, the last one or two (according to availability) plasma samples prior to diagnosis were retrospectively analysed by quantitative CMV PCR. In patients with more than one CMV diagnosis at different time points during life or at autopsy, plasma samples taken prior to the first CMV diagnosis were tested. Similarly, for patients without CMV disease, the last one or two plasma samples before death were tested. The samples were analysed by COBAS AMPLICOR CMV Monitor test (Roche Molecular System, Branchburg, NJ) according to the manufacturer's recommendations. One low-positive control, one high-positive control, and one CMV-negative control were processed with each batch of samples. The linear range of this assay is between 400 and 100 000 CMV DNA copies/mL plasma.

A total of 82 plasma samples were tested before diagnosis in patients with CMV disease, and 127 samples were analysed from patients without CMV disease. Median time interval between the last plasma sample and the date of the first (or only) CMV diagnosis (alive or post-mortem) was 69 days. Median time interval between the last plasma sample and date of death in patients not developing CMV disease at any time was 79 days. This difference was not statistically significant. For patients first diagnosed alive with CMV disease, the median time interval between the last sample and diagnosis was 64 days, as compared to 70 days for patients first diagnosed at autopsy (statistically not significantly different). Among 29 patients with CMV disease and 55 patients without CMV disease from whom two samples were tested, median time intervals between the two tests were 77 days and 90 days, respectively, again not statistically different.

The CMV quantitative PCR results were used to study sensitivity, specificity, PPV and NPV of the test with different cut-off values for CMV viral load, and with viraemia in one versus two samples.

CD 4 cell counts were available from electronic records and had been measured in blood samples taken on the same day that plasma had been taken for later quantitative CMV PCR.

SPSS statistical software version 13.0 (SPSS Inc. New York, 2004) was used to calculate Spearman rank correlation between CD4 cell counts and CMV viral load. Statistical differences between groups were calculated using non-parametric tests (Mann-Whitney and chi-square test as appropriate). Logistic regression was performed using presence or absence of CMV disease as dependent variable and last CD4 cell count (above or below 100/mm^3^, last CMV viral load (log transformed), HAART (yes or no), time in days between last plasma sample or outcome defined as CMV disease or death without CMV disease, sex, and age at time of death as independent variables. Confidence Interval Analysis statistical software (Wilson's method) was used to calculate sensitivity, specificity, PPV and NPV with confidence intervals. All confidence intervals (CI) are 95% CIs. The significance level was set at 5%.

## Results

Twenty-seven of 53 patients with CMV disease (51%) and 10 of 72 patients without CMV disease (13%) had detectable viraemia in at least one sample (p < 0.001). Results of CMV quantitative PCR from the last plasma sample are shown in Table 2. There was significantly higher CMV viral load in patients with CMV disease compared to patients without CMV disease (p < 0.001). In 25 patients with CMV disease and 8 patients without CMV disease who had viraemia in the last sample, median CMV viral loads were 3420 (range 392–261 000) and 1705 (range 277–9620) DNA copies/mL, respectively (statistically not significantly different). Table 2 also shows the number of cases with detectable CMV viraemia according to the number of samples tested.

**Table 2:** Results of CMV quantitative PCR in patients with and without CMV disease

Results of CMV PCR assay	Number of patients	
	with CMV disease	without CMV disease
	N = 53	N = 72
Viral load (CMV DNA copies/mL) (last samples)		
Below limit of detection	28	64
Viraemia^a^	25	8
≤1 999	9	5
2 000–9 999	7	3
≥10 000	9	0
		
CMV DNA detection^a ^by no. of tested samples before diagnosis		
One sample	24	17
Not detected in any sample	13	15
Detected in one sample	11	2
Two samples	29	55
Not detected in any sample	13	47
Detected in one sample	9	8
Detected in both samples	7	0

^a^Any viraemia above limit of detection.

Results of plasma CMV quantitative PCR in HIV patients with and without CMV disease by different viral load cut-offs and number of tested samples.

Among 16 patients diagnosed alive with CMV disease, 7 (44%) had detectable CMV viraemia in the last sample before diagnosis (median 1810 DNA copies/mL among viraemic cases). Among 37 patients first diagnosed with CMV disease at autopsy, 18 (49%) had CMV viraemia in the last sample (median 4430 DNA copies/mL among viraemic cases). There was no significant difference in the proportion of cases with viraemia (sensitivity) or level of viraemia between these two groups of patients.

In 37 patients first diagnosed with CMV disease at autopsy, 14 of 20 patients with CMV disease in more than one organ had detectable CMV viremia in the last sample (median 7035 copies/mL among viraemic cases), compared to 4 of 17 patients with CMV disease in a single organ, (median 2680 DNA copies/mL among viraemic cases). In other words, sensitivity of the test was 70% for cases with multi-organ disease and 24% for cases with single organ disease (p = 0.005). However, among viraemic cases there was no statistically significant difference in CMV viral load.

Results from Table 2 were used to calculate the corresponding sensitivity, specificity, PPV and NPV with increasing CMV viral load cut-off, and with one or two samples with detectable viraemia in individual patients (Table 3). The viral load cut-off is defined as the lowest level of viraemia which is considered a positive result for diagnostic purposes. Sensitivity and NPV were generally low under a wide range of assumptions. Specificity and PPV were generally higher, and were improved further by raising the viral load cut-off. By setting the cut-off at 10 000 CMV DNA copies/mL plasma, a specificity of 100% was attained, but at the expense of sensitivity which fell to 17%. Also, a specificity and PPV of 100% was achieved by defining a positive test as CMV viraemia in two samples.

**Table 3:** Sensitivity, specificity, positive and negative predictive values of plasma CMV quantitative PCR

Results of CMV PCR assay	Sensitivity (%) (95% CI)	Specificity (%) (95% CI)	PPV (%) (95% CI)	NPV (%) (95% CI)
Viral load (CMV DNA copies/mL (last samples)				
Viraemia^a^	47 (34–60)	89 (80–94)	76 (59–87)	70 (60–78)
≥2 000	30 (20–44)	96 (89–99)	84 (62–95	65 (56–74)
≥10 000	17 (9–29)	100 (95–100)	100 (70–100)	62 (53–70)
				
CMV DNA detection^a ^by no. of tested samples before diagnosis				
One sample				
CMV DNA detected	46 (30–65)	90 (69–97)	85 (58–96)	57 (39–73)
Two samples				
CMV DNA detected in at least one sample	55 (38–72)	86 (74–92)	67 (47–82)	78 (66–87)
				
CMV DNA detected in both samples	24 (12–42)	100 (94–100)	100 (65–100)	70 (61–80)

^a^Any viraemia above limit of detection.

Sensitivity, specificity, positive and negative predictive value of plasma CMV quantitative PCR in the diagnosis of CMV disease in HIV patients by different viral load cut-offs and number of tested samples.

**Figure 1:**
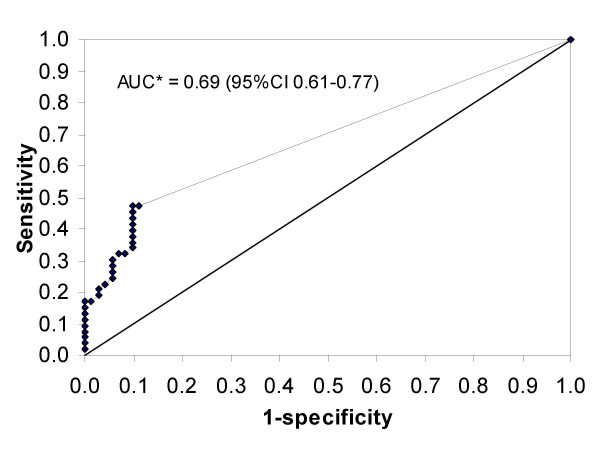
**CMV quantitative PCR results expressed as ROC curve**. Receiver operating characteristic (ROC) curve based on quantitative CMV PCR results in the last available plasma sample prior to the first diagnosis of CMV disease (alive or at autopsy) or death without CMV disease. *AUC = area under the curve.

The proportion of cases with CMV disease that have a positive tests (sensitivity) plotted against the proportion of cases without CMV disease that have a positive test (1-specificity) for all measured values of CMV DNA viraemia in the last plasma sample are shown as a receiver operating characteristic (ROC) curve in Figure 1. Using the lowest detectable level of viraemia as cut-off, a maximum sensitivity of 47.2% was attained. However, specificity of 1 (100%) (1-specifity = 0) was attained with a cut-off of 10 000 CMV DNA copies/mL as mentioned above. The diagnostic accuracy of the test, expressed by the area under the curve, was 0.69. This area corresponds to the probability that a random person with the disease has a higher CMV viral load than a random person without CMV disease.

CMV viraemia was negatively correlated with CD4 cell counts (r_S _= -0.24 (p < 0.01). In the multivariable logistic regression model low CD4 cell counts and high CMV viral load were the only statistically significant positive predictors of CMV disease.

## Discussion

With the exception of retinitis, end-organ manifestations of CMV disease in HIV-infected individuals have unspecific clinical characteristics, and histopathological confirmation is required for definite diagnosis. Because biopsy of affected organs may be difficult to obtain, there is a need for microbiological diagnostic methods that can be used on readily available specimens. A variety of tests to detect CMV nucleic acids have been evaluated in different patient populations. To our knowledge, no previous tests have been evaluated in relation to autopsy findings, an important shortcoming, as histological verification of CMV disease is often first obtained at autopsy. The main strength of this study is that it explores the utility of quantitative PCR in the diagnosis of CMV disease in HIV-infected individuals with available autopsy results. Also, it includes one of the highest reported number of cases of CMV disease in HIV-infected patients evaluated with CMV PCR.

Several qualitative and quantitative PCR methods for CMV in plasma in various patient populations have demonstrated sensitivity in the range 35–93% [13,18-20,23,26,27]. In our study, maximum sensitivity of 47% for the total number of cases, using a cut-off at the limit of detection, was in the lower end of this range. In a small previous study on HIV-infected individuals using COBAS AMPLICOR CMV Monitor, sensitivity was 92% [27]. The relatively low sensitivity in our study could be due to samples in many cases having been taken as part of routine monitoring and therefore too long, a median of 69 days, before development of CMV end-organ disease. However, CMV viraemia is known to often precede development of CMV disease by several months [2,13,17]. Also, in contrast to previous studies, most of our cases of CMV disease were first diagnosed at autopsy, and it is possible that these post-mortem manifestations are associated with a shorter time with viraemia and/or lower level of viraemia. Interestingly, our results show that sensitivity was significantly higher for patients with CMV disease in more than one organ compared to patients with single organ disease.

Previous studies of CMV disease diagnosed before death have yielded specificities in the range 47–100% [13,18-20,23,26,27]. Our study shows specificity in the upper range (89%), with only 8 of 72 (11%) cases having detectable CMV viraemia but no diagnosis of CMV disease (false positive tests). Studies that do not include autopsy results may underestimate the specificity, as CMV end-organ disease may be a difficult clinical diagnose to make.

Our data were also analysed with regard to detection of CMV viraemia in one versus two plasma samples taken at different times. We found that cases with detectable CMV viraemia in both samples all developed CMV disease. Thus, by requiring confirmation in a second test if the first was positive, a specificity of 100% was attained, but at the cost of lower sensitivity.

From a clinician's point of view, the predictive value of a positive and negative test result has greater practical implications than do sensitivity and specificity. The predictive value of any test is generally dependent on the prevalence of the disease in question in the study population, NPV decreasing and PPV increasing with increasing prevalence. Previous studies have reported NPV in the range 80–95% [20,23,26,27], which is somewhat higher than in our study. The prevalence of CMV disease in our study was comparatively high – 42% (53 of 125 patients) – thus contributing to a low NPV. For the same reason, the PPV demonstrated in our study was among the highest reported. In our patient population at high risk of CMV disease we attained a PPV of 100% with a cut off-value of 10 000 CMV DNA copies/mL or a requirement for viraemia in two consecutive tests. Studies that do not include autopsy results may underestimate the proportion of cases with CMV disease, resulting in an overestimation of the NPV and an underestimation of the PPV.

The study covers a period both before and after HAART became standard of care. Even in patients on HAART, antiretroviral treatment was suboptimal for many, probably due to HIV resistance having developed after previous ineffective therapy with single drugs, or combination of two antiretrovirals. This may affect the external validity of our results in the diagnosis of CMV disease in HIV-infected patients who have access to more effective HAART today and, as a result, have lower risk of CMV disease. However, results from our study are likely to be relevant for patients who interrupt treatment, who fail to respond to antiretroviral therapy, or who are diagnosed too late for effective HIV therapy to be initiated.

This was a retrospective study in which the time intervals varied considerably between the last plasma sample and CMV diagnosis (alive or at autopsy), or between the last plasma sample and death in cases without CMV disease, as samples were taken as part of routine monitoring of the patients rather than on clinical suspicion. It is likely that the sensitivity and PPV of CMV PCR will be higher in a setting where plasma is analysed at a time of clinical suspicion of CMV end-organ disease, rather than as part of routine monitoring of patients.

The availability of more than one plasma sample from individual patients at relevant time points was limited, but a high positive predictive value for CMV disease was found for patients with CMV viraemia in two consecutive tests with a median time interval of between two and three months. However, our data do not allow us to conclude when plasma CMV quantitative PCR should be performed, or how soon a patient should be retested after a positive test result.

In solid organ transplant patients and allogenic bone marrow transplantation, routine monitoring of virological markers for cytomegalovirus and administration of pre-emptive therapy has been shown to significantly reduce the risk of CMV disease [30,31], and post-transplant monitoring of CMV viraemia by PCR or other methods is used in many centres. Among HIV-infected individuals the value of pre-emptive therapy is less well documented, but monitoring of high risk patients for this purpose is recommended in French guidelines [11].

Many studies have shown that the risk of CMV disease sharply increases when CD4 cell counts fall below 100/mm^3 ^[2,3,32]. We suggest that HIV infected patients with low CD4 cell counts who have clinical features suggestive of CMV disease, should have plasma tested for CMV by quantitative PCR as part of the diagnostic procedure. In patients with repeated viraemia or viraemia above 10 000 CMV DNA copies/mL, pre-emptive therapy should be considered after a careful clinical examination for signs and symptoms of CMV end-organ disease. However, our findings need to be validated in prospective studies on patients living with HIV today.

## Conclusion

Using CMV quantitative PCR, the sensitivity and NPV for CMV disease were low in this study. Our results indicate that in a population at high risk of CMV disease quantitative CMV PCR is best used to rule in, rather than to rule out CMV disease. Although CMV disease presently is relatively uncommon in HIV-infected patients who have access to antiretroviral therapy, these results may have implications for diagnosis of CMV disease in patients with low CD4 cell counts due to late HIV diagnosis or failure of antiretroviral therapy.

## Competing interests

None of the authors declare any conflict of beyond those declared in the acknowledgements regarding the manufacturer of the diagnostic test.

## Authors' contributions

All authors have made substantial contributions to conception and design of the study, have been involved in writing and revising the manuscript critically, have read and given approval of the final version of the manuscript submitted for publishing. ABB has had main responsibility for analysing the data and collecting clinical information about the cases. AKG has had main responsibility for the pathological diagnosis of CMV disease in tissue samples. MHP has been responsible for analysis of CMV viraemia by quantitative CMV PCR in plasma samples. All authors read and approved the final manuscript.

## Pre-publication history

The pre-publication history for this paper can be accessed here:


